# Dietary Factors and Tinnitus among Adolescents

**DOI:** 10.3390/nu12113291

**Published:** 2020-10-27

**Authors:** Milena Tomanic, Goran Belojevic, Ana Jovanovic, Nadja Vasiljevic, Dragana Davidovic, Katarina Maksimovic

**Affiliations:** 1 Institute of Hygiene and Medical Ecology, Faculty of Medicine, University of Belgrade, 11000 Belgrade, Serbia; goran.belojevic@med.bg.ac.rs (G.B.); ana.s.jovanovic@med.bg.ac.rs (A.J.); nadja.vasiljevic@med.bg.ac.rs (N.V.); dragana.davidovic@med.bg.ac.rs (D.D.); 2 Faculty of Medicine, University of Belgrade, 11000 Belgrade, Serbia; katarinamaksimovic00@gmail.com

**Keywords:** adolescents, dietary factors, tinnitus

## Abstract

The number of people suffering from constant tinnitus is ever-increasing and has spread to all age groups, including adolescents. The etiology of tinnitus is multifactorial, but dietary factors have been rarely investigated. The purpose of this study is to examine the relationship between dietary factors and constant tinnitus among adolescents from an urban environment. A population-oriented cross-sectional study was carried out during the 2019/2020 school year in 12 Belgrade secondary schools. There were 1287 school children aged from 15 to 19 years who participated in the study. There were 1003 respondents who completed a questionnaire on tinnitus (response rate 77.9%; 31% male). We used the standardized Tinnitus Screener questionnaire and a food frequency questionnaire specially designed for this study and adapted to Serbian adolescents. A logistic regression analysis revealed a strong negative correlation between fresh vegetables and fruits and tinnitus presence. On the other hand, the risk of constant tinnitus increased with the increased intake of white bread, carbonated beverages, and fast food. In conclusion, we show that fresh fruit and vegetable intakes may be negatively related to tinnitus frequency, while sweetened sodas, fast food, and white bread may raise the odds for tinnitus.

## 1. Introduction

Tinnitus is a conscious perception of sound in the absence of an appropriate external sound stimulus [1]. It is classified under the code H93 in the International Classification of Diseases, Tenth Revision, Clinical Modification Diagnosis Code classification [2]. Tinnitus can be perceived as constant (always heard in a quiet environment), intermittent = vacillates between “present” and “not present” on a regular basis, e.g., daily or weekly), occasional (tinnitus that is experienced on an irregular basis, e.g., “monthly or yearly”), and temporary (linked to some event (e.g., noise or ototoxicity) and lasts a period of time following the event and then subsides [3]. There is no consensus on the period of time that defines chronic tinnitus; according to some authors [3], it should last at least six months.

In earlier years, tinnitus was usually regarded as a side effect of hearing impairment, typically among the elderly [4]. However, the percentage of people facing the problem of phantom sound has increased and spread to all age groups, including adolescents.

Tinnitus occurs at least once in a lifetime in a third of people, but in 10–15% of the adult population, it is chronic [5]. In children and adolescents, at the age of 5–19 years, the range of tinnitus prevalence is much wider (from 5% to 75%) depending on how the term tinnitus is defined in a study [6].

Different studies have shown different attitudes of children towards tinnitus. On the one hand, it has been found that children see tinnitus as something normal or not as a serious health problem [7], but other studies have shown that tinnitus in children creates fear, tension, and anxiety at this age [8,9,10,11]. Although children and adolescents know how to accurately describe tinnitus, they rarely spontaneously complain of it [12].

Despite the growing number of studies on tinnitus, its etiology is still vague, and its occurrence has been associated with a large number of personal and environmental factors (hearing impairment, Meniere’s disease, otosclerosis, noise, ototoxic drugs, etc.) [13].

There is a lack of scientific data on the possible relationship between diet and tinnitus. To our knowledge, only four population-based studies have been published in this field, and all of them were performed on an adult and/or elderly population [14,15,16,17]. In the first population study on diet–tinnitus association that was performed among UK adults, it was found that the odds of tinnitus were increased with a higher intake of fruits and vegetables, whole grain bread, and dairy avoidance. On the other hand, tinnitus was reduced with egg avoidance, fish consumption and caffeinated coffee consumption [14]. In another study on a British adult population, a negative relationship with the frequency of tinnitus was found for vitamin B12, while calcium, iron, and fat raised the odds for the onset of tinnitus [15]. Spankovich et al. performed a population study of diet and tinnitus in the United States using the healthy eating index. They found that, with a healthier diet (a number of servings of meat, dairy, fruit, vegetables and grains close to the recommended number) the odds of persistent tinnitus were decreased [16]. Lee and Kim conducted a population-oriented study of tinnitus in Korean adults. They revealed that the risk factors for tinnitus were a reduced intake of water, protein, riboflavin and niacin [17]. In a single study that only partially investigated a diet-tinnitus relationship within other risk factors among adolescents, the authors used neither a standardized tinnitus questionnaire nor a detailed food frequency questionnaire [18].

In our opinion, there is a need to investigate constant tinnitus, as it is the most serious form of tinnitus. We also support using standardized questionnaires such as the Tinnitus Screener questionnaire [3]. The scientific challenge is that, unlike adults, adolescents often ignore tinnitus and find it difficult to decide to visit a doctor [19].

The aim of this study is to examine the relationship between dietary factors and constant tinnitus among urban adolescents. We hypothesize that the intake of some food items is significantly related to the onset of tinnitus.

## 2. Materials and Methods

The Ethics Committee of the Faculty of Medicine University of Belgrade approved this study (Decision No. 1550/XI-38 on 11/28/2019). An informed consent was signed by all participants, and additional parental informed consent was obtained for children younger than 18 years.

### 2.1. Study Design

We performed a population-oriented cross-sectional study during the 2019/2020 school year in 12 Belgrade high schools. The respondents completed questionnaires during a class.

### 2.2. Study Sample

There were 1287 students who participated in the study (37.4% male). A questionnaire was completed by 1003 students (31.4% male). The response rate was 77.9%. No significant difference in gender, socioeconomic status, and parental education was found between respondents and nonrespondents.

### 2.3. Questionnaires

The first set of questions (Supplementary File S1) was related to gender, age, socioeconomic status, parental education, noise annoyance, sleep, listening to music through earphones, going out to noisy places, coffee, smoking, drug use, sedation, head injury, and associated diseases.

### 2.4. Data Were Collected on Gender and Age

#### 2.4.1. Socioeconomic Status

Respondents were asked to answer whether their material needs were fulfilled. The offered answers were “No” and “Yes”.

#### 2.4.2. Parental Education

The offered answers were “High school” and “College”.

#### 2.4.3. Noise Annoyance

Respondents were asked to answer how much noise had bothered, disturbed or hindered them in the previous 12 months, while they had been at home and/or at school. The offered answers were “Little”, “Moderate” and “Much”.

#### 2.4.4. Sleep

Respondents were asked to answer how many hours on average they slept at night. The offered answers were: “Less than six hours”, “Between six and eight hours” and “More than eight hours”.

#### 2.4.5. Music Through Earphones

Respondents were asked how many hours they used earphones to listen to the music and other loud sounds. The offered answers were “Less than one hour”, “From one to three hours” and “More than three hours”.

#### 2.4.6. Going Out to Noisy Places

Participants were asked how often they went out to places with loud music. The offered answers were “Three times a month or less” and “Once a week or more”.

#### 2.4.7. Smoking

Smoking habits were investigated with the question “Do you smoke, or have you ever smoked?” The offered answers were “No” and “Yes”.

#### 2.4.8. Drug Use

Respondents were asked if they use drugs, with the offered answers “No” and “Yes”.

#### 2.4.9. Sedatives

Respondents were asked “Are you taking tranquilizers?”, and the offered answers were “No” and “Yes”.

#### 2.4.10. Head Injury

Respondents were asked if they had ever experienced a severe head injury. Possible answers were “No” and “Yes”.

#### 2.4.11. Associated Diseases

Data on comorbidities were obtained through the following separate questions: “Have you ever had: an ear infection, anxiety or depression, high blood pressure, thyroid problems, sinusitis, or anemia?” Possible answers were “No” and “Yes”.

### 2.5. Tinnitus Screener

The second part of questionnaire that respondents answered was the standardized Tinnitus Screener questionnaire [3]. For the purposes of this study, only constant tinnitus was taken as a binary health outcome.

### 2.6. Dietary Assessment

In the third part of the questionnaire, there was a food frequency inventory that we designed following the basic framework from the 2012 Youth Adolescent Food Frequency Questionnaire [20] and adapted for Serbian adolescents. The questions on food intake were semi–quantitative (e.g., number of tablespoons or portions). We asked about the intake of fruits; vegetables; fish; eggs; milk; bread; French fries; fast food; supplements rich in sugars; salts; and fats (ketchup, mustard, mayonnaise, butter and margarine); snacks; sodas; energy drinks; beer; wine; spirits; artificial sweeteners; food salting; and multivitamin supplements.

### 2.7. Statistical Analysis

In a descriptive statistic, we used a percentage calculation. In an inferential statistic, the distribution of tinnitus by gender was examined with the chi-square test. The association between personal and dietary factors as independent variables and constant tinnitus as a dependent variable was investigated with a univariate logistic regression.

The results were analyzed statistically using IBM SPSS ver. 21.0 (IBM North America, New York, NY 10022, USA).

## 3. Results

We found that 134 (13.4%) respondents had tinnitus; it was more frequent among boys (16.8%) (53/315) than among girls (11.8%) (81/688) (chi-square test, *p* < 0.029) (Table 1). Tinnitus increased with age and socioeconomic status. On the other hand, a higher parental education was negatively related to tinnitus frequency. However, these relationships were not statistically significant (Table 1).

Concerning personal confounding variables, female gender and ear infections showed a significant negative association with tinnitus.

We found a positive relationship between tinnitus and drug use, going out to noisy places, hypertension, using earphones, head injury, anxiety or depression, thyroid gland disorder, and smoking. Tinnitus was negatively related to sedatives use, anemia, sinus infection, exposure to noise at home, night sleep duration and exposure to noise at school, but these relationships were statistically insignificant (Table 2).

Concerning the diet–tinnitus relation, a strong negative correlation was found for fresh vegetables and fruits. On the other hand, the risk for onset of constant tinnitus increased with the increased intake of white bread, carbonated beverages and fast food (Table 3).

A statistically insignificant positive relationship was found between tinnitus and salting food, the intake of French fries, coffee, water, margarine, snacks, pastries from the bakery, ketchup, mustard, mayonnaise, supplements in the form of multivitamin complexes and fish. On the contrary, tinnitus was insignificantly and negatively related to the intake of eggs, milk, wine, energy drinks, artificial sweeteners, beer and spirits (Table 3).

## 4. Discussion

We show that a higher intake of fresh fruits and vegetables is negatively related to the onset of constant tinnitus among adolescents. Fruits, vegetables, and legumes are major sources of minerals, vitamins and antioxidants (vitamins C, vitamin A, chlorophyll, potassium, flavonoids, dietary fiber carotenoids, folate, curcumin) that stop the spread of chain reactions and free radicals damage [21,22,23].

Free radicals and reactive oxygen species are molecules that may disturb the homeostasis of the organism. The imbalance of the endogenous antioxidant system and free radicals and reactive oxygen species levels can cause damage to cell membranes, cytosols and mitochondria [24,25]. The excessive accumulation of free radicals in the sensory epithelium of the cochlea spiral ganglion neurons and cells of stria vascularis plays an important role in the development of damage to auditory cells [25] and the subsequent development of symptoms, including tinnitus. Therefore, a diet rich in antioxidants may be a potential protective factor against tinnitus.

Our findings related to fruits and vegetables are in accordance with the results of previous studies on the relationship between diet and constant tinnitus in adults [14,17,26]. Contrary to this view, in a study by McCormack et al. (2014) in adults [15], it was found that increasing fruit and vegetable intakes moderately increased the odds for persistent tinnitus but was not related to transient tinnitus.

On the other hand, we show that white bread, fast food, and sweetened sodas raise the odds for tinnitus among adolescents. Similar to our study, whole grain bread was found to be protective against tinnitus in adults in a large British study by McCormack et al. (2014) [15]. Spankovich et al. (2017) [16] found that a healthy diet (indexed by a Healthy Eating Index (HEI) score, United States Department of Agriculture 1995 [27]) was associated with a reduced risk of developing persistent tinnitus among adults. The term “healthier HEI scores” referred to the intake of larger quantities of fruits and vegetables, as well as whole grain cereals and lower sugar intakes. White bread, fast food, and sweetened sodas may be risk factors for tinnitus due to their high glycemic index; consequent hyperinsulinemia might cause microvascular complications and inner ear diseases and tinnitus [28].

Our finding of more frequent tinnitus in boys compared to girls is not in agreement with the results of previous studies, in which tinnitus was about two times more frequent among girls (compared to boys) [29,30]. However, our gender distribution of tinnitus is similar to the findings of studies among adult populations [31].

A possible explanation for our findings may be significantly more frequent nights out at places with loud music (chi-square, *p* = 0.005), more frequent head injuries (*p* < 0.001) and frequent exposure to explosions near ears (*p* < 0.001). Additionally, in our study, we found a higher frequency of narcotics use (*p* = 0.031) and alcohol consumption (*p* = 0.009) among boys compared to girls, as well as the tendency to play games of chance (*p* < 0.001), which can be related to great stress, which is also considered as an important factor for the development of tinnitus in general.

Our finding on the negative association of ear infections with tinnitus is also not in agreement with the results of other studies [32,33]. Maybe the explanation for this outcome is in fact that those who had ear infections in childhood in later life tend to avoid exposure to noise, alcohol, active and second–hand smoke, and other risk factors for tinnitus [34,35].

### Strengths and Limitations of the Study

The strength of this study is that we used a standardized questionnaire for tinnitus and focused exclusively on a constant tinnitus, thus minimizing a selection bias.

The weakness of the study is that we did not perform a laboratory food analysis. Second, neither clinical examinations nor hearing tests were performed. Third, we did not conduct anthropometric measurements (body weight, height, body fat, and waist circumference). Fourth, we focused on the children of an urban setting; it is questionable whether the risk factors for tinnitus in suburban and rural areas would be the same.

## 5. Conclusions

We show that the intake of fresh fruits and vegetables might be an independent dietary factor that is negatively related to the onset of tinnitus among adolescents. Food that may raise the odds for tinnitus among young people are sweetened sodas, fast food and white bread. For causal interpretations of these relationships, we suggest interventional studies.

## Figures and Tables

**Table 1:** Sociodemographic characteristics of the investigated children.

	Constant Tinnitus						
	Valid *n*	No		Yes		Missing	*p* *
		Count		Count			
		% Within Specific. Group	% of Total	% Within Specific Group	% of Total		
Gender	1003 (77.9%)					284 (22.1)	0.029
Male	315 (31.4%)	262		53			
		83.2%	26.1%	16.8%	5.3%		
Female	688(68.6%)	607		81			
		88.2%	60.5%	11.8%	8.1%		
Age	1003 (77.9%)					284 (22.1%)	0.726
15	37 (3.7%)	34		3			
		91.9%	3.4%	8.1%	0.3%		
16	203 (20.2%)	180		23			
		88.7%	17.9%	11.3%	2.3%		
17	258 25.7%	222		36			
		86.0%	22.1%	14.0%	3.6%		
18	193 19.2%	166		27			
		86.0%	16.6%	14.0%	2.7%		
19	312 31.1%	267		45			
		85.6%	26.6%	14.4%	4.5%		
Perception of Family Income	1000 (77.7%)					287 (22.3%)	0.251
Fulfilled needs	922 (92.2%)	797		125			
		86.4%	79.7%	13.6%	12.5%		
Unfulfilled needs	78 (7.8%)	71		7			
		91.0%	7.1%	9.0%	0.7%		
Parental education							
Father (High school)	991 (77.0%)	384		68		296 (23.0%)	0.170
		85.0%	38.7%	15.0%	6.9%		
Father (College)	991 (77.0%)	474		65		296 (23.0%)	
		87.9%	47.8%	12.1%	6.6%		
Mother (High school)	994 (77.2%)	424		74		293 (22.8%)	0.170
		85.1%	42.7%	14.9%	7.4%		
Mother (College)	994 (77.2%)	437		59		293 (22.8%)	
		88.1%	44.0%	11.9%	5.9%		

* Pearson chi-square.

**Table 2:** The univariate logistic regression between personal factors and tinnitus among adolescents (*n* = 1003).

	Constant Tinnitus		
	Odds Ratio	95% Confidence Interval	*p*-Value
Potential confounders			
Gender (Male = 1; Female = 2)	0.660	(0.453–0.960)	0.030
Exposure to noise at home (1 = Little; 2 = Moderate; 3 = Much)	0.860	(0.629–1.177)	0.346
Exposure to noise at school (1 = Little; 2 = Moderate; 3 = Much)	0.892	(0.708–1.123)	0.331
Night sleep duration (per hour) (1 = ≤6 h; 2 = 6–8 h; 3 = ≥8 h)	0.874	(0.636–1.200)	0.405
Using earphones (per hour) (1 = ≤1 h; 2 = 1–3 h; 3 = ≥3 h)	1.182	(0.904–1.545)	0.222
Going out to noisy places (1 = 3 times a month or less; 2 = once a week or more)	1.489	(0.963–2.301)	0.073
Smoking status (0 = No; 1 = Yes)	1.044	(0.658–1.657)	0.854
Drugs use (0 = No; 1 = Yes)	2.461	(0.964–6.402)	0.065
Sedatives use (0 = No; 1 = Yes)	0.768	(0.343–1.720)	0.520
Head injury (0 = No; 1 = Yes)	1.141	(0.789.–1.651)	0.484
Ear infections (0 = No; 1 = Yes)	0.626	(0.406–0.964)	0.034
Anxiety or depression (0 = No; 1 = Yes)	1.077	(0.682–1.702)	0.750
Hypertension (0 = No; 1 = Yes)	1.234	(0.566–2.689)	0.596
Thyroid gland disorder (0 = No; 1 = Yes)	1.051	(0.779–1.418)	0.747
Sinus infection (0 = No; 1 = Yes)	0.846	(0.537–1.331)	0.469
Anemia (0 = No; 1 = Yes)	0.776	(0.177–3.400)	0.737

**Table 3:** The univariate logistic regression between dietary factors and constant tinnitus among adolescents (*n* = 1003).

	Constant Tinnitus		
Food Type Intake	Odds Ratio	95% Confidence Interval	*p*-Value
Supplements in the form of multivitamin complexes (0 = No; 1 = Yes)	1.023	(0.664–1.574)	0.919
Sodas (0 = Never; 1 = Weekly; 2 = Every day; 3 = Few times daily)	1.249	(1.014–1.538)	0.036
Energy drinks (0 = Never; 1 = Weekly; 2 = Every day; 3 = Few times daily)	0.906	(0.636–1.290)	0.583
Daily coffee intake (0 = None; 1 = One or two; 2 = Thee or more)	1.373	(0.944–1.998)	0.097
Daily water intake (1 = <1 L; 2 = Between 1–2 L; 3 = ≥More than 2 L)	1.208	(0.947–1.542)	0.128
Beer (0 = Never; 1 = <1 L; 2 = More than 1 L)	0.923	(0.700–1.216)	0.568
Wine (0 = Never; 1= <1/2 L; 2 = More than l/2 L)	0.898	(0.672–1.200)	0.467
Spirits (0 = Never; 1= <1/2 L; 2 = More than l/2 L)	0.985	(0.762–1.274)	0.911
Milk (0 = Don’t drink a milk; 1 = Drink milk with <1% m. fat; 2 = Drink milk with >1% m. fat)	0.897	(0.730–1.102)	0.301
Margarine (0 = Monthly or less; 1 = Weekly; 2 = Every day)	1.204	(0.889–1.631)	0.230
Fast food (1 = Monthly or less; 2 = Weekly; 3 = Every day)	1.355	(1.011.–1.816)	0.042
Fish (1 = Monthly or less; 2 = Weekly; 3 = Every day)	1.004	(0.699–1.442)	0.984
Eggs (1 = Monthly or less; 2 = Weekly; 3 = Every day)	0.733	(0.525–1.023)	0.067
Ketchup, mustard, mayonnaise (1 = Monthly or less; 2 = Weekly; 3 = Every day)	1.043	(0.815–1.335)	0.737
Artificial sweeteners (1 = Monthly or less; 2 = Weekly; 3 = Every day)	0.920	(0.667–1.270)	0.614
Type of bread (1 = Whole grain bread; 2 = White bread)	1.715	(1.115–2.638)	0.014
Pastries from the bakery (1 = Monthly or less; 2 = Weekly; 3 = Every day)	1.051	(0.779–1.418)	0.747
French fries (1 = Monthly or less; 2 = Weekly; 3 = Every day)	1.380	(0.989–1.926)	0.058
Fresh fruits (1 = Monthly or less; 2 = Weekly; 3 = Every day)	0.637	(0.462–0.879)	0.006
Fresh vegetables (1 = Monthly or less; 2 = Weekly; 3 = Every day)	0.244	(0.173–0.343)	<0.001
Snacks (1 = Monthly or less; 2 = Weekly; 3 = Every day)	1.124	(0.806–1.568)	0.490
Salting food (0 = No; 1 = Yes)	1.399	(0.874–2.240)	0.162

## Supplementary Materials

The following are available online at https://www.mdpi.com/2072-6643/12/11/3291/s1, Supplementary File S1: Questionnaires.
