# Supplementary material for: Dietary Factors and Tinnitus among Adolescents
Source: Nutrients. 2020 Oct 27;12(11):3291. doi: 10.3390/nu12113291 (PMC7693091; doi:10.3390/nu12113291)
Supplement: Supplementary file 1 [file nutrients-12-03291-s001.pdf]

QUESTIONNAIRES:

Personal factors

1. **Gender:** 1. Male 2. Female
2. **Date of your birth:** ___________
3. **Do you think that your parents can fulfill all your financial needs?**
   0. No 1. Yes
4. **Paternal education:**
   1. High school 2. College
5. **Maternal education:**
   1. High school 2. College
6. **Thinking about the last 12 months, while staying at home, how much noise has bothered, disturbed or hindered you?**
   1. Little 2. Moderate 3. Much

7. **Thinking about the last 12 months, while being at school, how much noise has bothered, disturbed or hindered you?**
   1. Little 2. Moderate 3. Much

8. **What is an average duration of your night sleep?**
   1. Less than 6 hours 2. Between 6 and 8 hours 3. More than 8 hours

9. **How many hours daily do you use earphones to listen to the music and other loud sounds?**
   1. Less than 1 hour 2. From 1 to 3 hours 3. More than 3 hours

10. **How often do you go out to places with loud music?**
    1.Three times a month or less 2. Once a week or more

11. **Do you smoke?** *
    0. No 1. Former smoker 2. Active smoker

12. **Do you use drugs?**
    0. No 1. Yes

13. **Do you take tranquilizers?**
    0. No 1. Yes

14. **Have you ever had a severe head injury?**
    0. No 1. Yes

15. **Have you ever had:**

Ear infection	0. No	1. Yes	Thyroid problems	0. No	1. Yes
Anxiety or depression	0. No	1. Yes	Sinusitis	0. No	1. Yes
High blood pressure	0. No	1. Yes	Anemia	0. No	1. Yes

* For the purpose of binary logistic regression, we had to merge the answers on active and former smoking into "Yes".

Tinnitus Screener

TINNITUS is ringing, buzzing, humming or other noises in your ears or head

During the PAST YEAR:

1. Have you experienced tinnitus lasting more than 2 - 3 minutes?
   NO: **STOP HERE** — **No Tinnitus**
   YES: **GO TO #2**

2. Have you experienced tinnitus for at least 6 months?
   NO: **GO TO #3** — **Acute Tinnitus**
   YES: **GO TO #3** — **Chronic Tinnitus**

3. In a quiet room, can you hear tinnitus?
   Always: **STOP HERE** — **Constant Tinnitus**
   Usually: **STOP HERE** — **Constant Tinnitus**
   Sometimes/Occasionally: **GO TO #4**

4. When you heard tinnitus this past year, was it caused by a recent event?
   (Examples: loud concert, head cold, allergies, some medications)
   NO: **GO TO #6**
   YES, Sometimes: **GO TO #5**
   YES, Always: **GO TO #5** — **Temporary Tinnitus**

5. Does your tinnitus seem to "come and go" on its own, in addition to being caused by a recent event(s)?
   NO: **STOP HERE** — **Temporary Tinnitus**
   YES: **GO TO #6**

6. Do you experience tinnitus on a:
   Daily or weekly basis: **STOP HERE** — **Intermittent Tinnitus**
   Monthly or yearly basis: **STOP HERE** — **Occasional Tinnitus**

Dietary Assessment

1. Are you taking any multivitamin?
0 =_No; 1 =_Yes
2. Do you drink sodas?
0 =_No; 1 =_Weekly; 2 =_Every day; 3 = _Few times daily
3. Do you drink energy drinks?
0 =_No; 1 =_Weekly; 2 =_Every day; 3 = _Few times daily
4. How many cups of coffee do you drink per day?
0 =_None; 1 =_One or two; 2 =_Thee or more
5. How much water do you drink per day?
1 =_<1l; 2 =_Between 1-2l; 3 =>_More than 2l
6. Do you drink beer?
0 =_No; 1 =_<1l per day; 2 =_More than 1l per day
7. Do you drink wine?
0 =_No; 1 =_<1/2l per day; 2 =_More than l/2l per day
8. Do you drink spirits?
0 =_No; 1 =_<1/2l per day; 2 =_More than l/2l per day
9. Do you drink milk and if you do what type?
0 =_No; 1 =_Yes, with <1% milk fat; 2 =_Yes, with >1% milk fat
10. How often do you eat margarine?
0 =_Monthly or less; 1 =_Weekly; 2 =_Every day
11. How often do you eat fast food?
1 =_Monthly or less; 2 =_Weekly; 3 =_Every day
12. How often do you eat fish?
1 =_Monthly or less; 2 =_Weekly; 3 =_Every day
13. How often do you eat eggs?
1 =_Monthly or less; 2 =_Weekly; 3 =_Every day
14. How often do you eat ketchup, mustard, mayonnaise?
1 =_Monthly or less; 2 =_Weekly; 3 =_Every day
15. How often do you use artificial sweeteners?
1 =_Monthly or less; 2 =_Weekly; 3 =_Every day
16. What type of bread do you eat?
1 =_Whole grain bread; 2 =_White bread
17. How often do you eat pastries from the bakery?
1 =_Monthly or less; 2 =_Weekly; 3 =_Every day
18. How often do you eat French fries?
1 =_Monthly or less; 2 =_Weekly; 3 =_Every day
19. How often do you eat fresh fruits?
1 =_Monthly or less; 2 =_Weekly or less; 3 =_Every day
20. How often do you eat fresh vegetables?
1 =_Monthly or less; 2 =_Weekly; 3 =_Every day
21. How often do you eat snacks?
1 =_Monthly or less; 2 =_Weekly; 3 =_Every day
22. Do you add salt to your food?
0 =_No; 1 =_Yes
